# DINOV2-FCS: a model for fruit leaf disease classification and severity prediction

**DOI:** 10.3389/fpls.2024.1475282

**Published:** 2024-12-06

**Authors:** Chunhui Bai, Lilian Zhang, Lutao Gao, Lin Peng, Peishan Li, Linnan Yang

**Affiliations:** ^1^ College of Big Data, Yunnan Agricultural University, Kunming, China; ^2^ Yunnan Engineering Technology Research Center of Agricultural Big Data, Kunming, China; ^3^ Yunnan Engineering Research Center for Big Data Intelligent Information Processing of Green Agricultural Products, Kunming, China

**Keywords:** DINOV2, deep learning, fruit disease recognition, semantic segmentation, smart agriculture

## Abstract

**Introduction:**

The assessment of the severity of fruit disease is crucial for the optimization of fruit production. By quantifying the percentage of leaf disease, an effective approach to determining the severity of the disease is available. However, the current prediction of disease degree by machine learning methods still faces challenges, including suboptimal accuracy and limited generalizability.

**Methods:**

In light of the growing application of large model technology across a range of fields, this study draws upon the DINOV2 visual large vision model backbone network to construct the DINOV2-Fruit Leaf Classification and Segmentation Model (DINOV2-FCS), a model designed for the classification and severity prediction of diverse fruit leaf diseases. DINOV2-FCS employs the DINOv2-B (distilled) backbone feature extraction network to enhance the extraction of features from fruit disease leaf images. In fruit leaf disease classification, for the problem that leaf spots of different diseases have great similarity, we have proposed Class-Patch Feature Fusion Module (C-PFFM), which integrates the local detailed feature information of the spots and the global feature information of the class markers. For the problem that the model ignores the fine spots in the segmentation process, we propose Explicit Feature Fusion Architecture (EFFA) and Alterable Kernel Atrous Spatial Pyramid Pooling (AKASPP), which improve the segmentation effect of the model.

**Results:**

To verify the accuracy and generalizability of the model, two sets of experiments were conducted. First, the labeled leaf disease dataset of five fruits was randomly divided. The trained model exhibited an accuracy of 99.67% in disease classification, an mIoU of 90.29%, and an accuracy of 95.68% in disease severity classification. In the generalizability experiment, four disease data sets were used for training and one for testing. The mIoU of the trained model reached 83.95%, and the accuracy of disease severity grading was 95.24%.

**Discussion:**

The results demonstrate that the model exhibits superior performance compared to other state-of-the-art models and that the model has strong generalization capabilities. This study provides a new method for leaf disease classification and leaf disease severity prediction for a variety of fruits. Code is available at https://github.com/BaiChunhui2001/DINOV2-FCS.

## 1 Introduction

In the contemporary globalized food supply chain, fruits occupy a pivotal position in the human diet. Fresh fruits, in particular, are highly esteemed for their alluring aroma and distinctive flavor (Wang et al., 2022). Fruit diseases represent a significant challenge for the fruit industry, accounting for significant economic losses annually. Timely identification of fruit diseases helps control infections and ensure optimal productivity (Khan et al., 2022). However, traditional fruit disease detection methods are susceptible to subjective judgement and experience differences of the inspector, leading to inconsistent and low accuracy of detection results (Khattak et al., 2021). Deep learning-based fruit disease detection methods not only significantly increase detection speed and accuracy, but also further optimise and enhance the ability of disease identification through continuous data accumulation and learning (Shoaib et al., 2023).

The development and implementation of autonomous plant disease detection has been made easier by the ongoing advancements in artificial intelligence technologies. A study (Atila et al., 2021) employed the EfficientNet model to identify diseases of plant leaves, with the objective of enhancing diagnostic accuracy and efficiency. By contrasting it with advanced convolutional neural network models, the study demonstrated that EfficientNet performs well in classifying plant leaf images, thereby validating its potential for automated diagnosis of plant diseases. The RIC-Net (Zhao et al., 2022) was developed on the foundation of the Inception and residual structure fusion models, with an enhanced Convolutional Block Attention Module (CBAM) integrated for the purpose of enhancing the efficacy of plant leaf disease classification. The DFN-PSAN (Dai et al., 2024) model demonstrated high performance in identifying diseases of plants through the application of weather data augmentation techniques on three datasets derived from real agricultural scenarios. The topic of plant disease identification has already reached a mature state of application for deep learning techniques.

Precisely determining the extent of plant diseases is vital from the standpoint of application. This is because the detection of disease severity assists farmers in making informed decisions to mitigate losses due to disease infection. A study (Zeng et al., 2020) created a HLB-infected citrus leaf image dataset, expanded the original training dataset with a deep convolutional generative adversarial network, and trained six different deep learning models to perform severity detection. A unique three-branch Swin Transformer classification network (TSTC) was designed in another study (Yang et al., 2023)to diagnose plant diseases and their severity independently and concurrently. However, these plant disease severity estimates are based on simple classification networks, which are less effective and weakly interpretable. In practice, calculating the percentage of leaf diseased area is a crucial step in assessing the severity of the disease (Madden et al., 2007). A study (Goncalves et al., 2021) trained six semantic segmentation models for the purpose of recognizing and estimating the severity of plant leaf diseases with an accuracy comparable to that of commercial software. This was achieved without the need to manually adjust the segmentation parameters or remove complex backgrounds from the images. Another study (Hu et al., 2021) employed a support vector machine to segment the lesion in order to better identify the disease and offered an elliptical restoration approach to fit and restore the whole size of the occluded or damaged tea leaves. Researchers presented a deep learning and fuzzy logic based approach to establish an automated technique for grapevine black measles disease identification and severity analysis (Ji and Wu, 2022). To address the problem of cucumber downy mildew, researchers proposed a two-stage segmentation framework to calculate the percentage of leaf disease area (Wang et al., 2021). The resulting accuracy of the disease severity classification was 92.85%. Nevertheless, all of these works have trained models just for a single plant disease, thus leading to limited generalization.

As computer vision technology advances, large vision models find extensive use in several domains. SAM (Kirillov et al., 2023), a powerful model designed for segmentation tasks, has been developed to achieve zero-sample migration to a variety of tasks through cueing engineering. It has demonstrated excellent performance on a range of image segmentation tasks, which has contributed to the advancement of the computer vision field. However, the considerable computational expense of SAM represents a significant obstacle to its broader deployment in industrial settings. FastSAM (Zhao et al., 2023), MobileSAM (Zhang et al., 2023a), and MobileSAMv2 (Zhang et al., 2023b) employ model parameter reduction and accelerate inference techniques to mitigate this challenge. DINO (Caron et al., 2021) employs a novel contrast learning method to enhance its visual generic representation. This method compares the features of the original image with those of a randomly cropped image, resulting in highly satisfactory outcomes. DINOv2 (Oquab et al., 2023) is a method for pre-training an image encoder on a large image dataset in order to obtain visual features with semantic meaning. These features can be employed for a diverse range of visual tasks without the necessity for further training to achieve performance levels comparable to those of supervised models. In the application of large vision models, MedSAM (Ma et al., 2024) was demonstrated to have significantly enhanced segmentation performance on medical images by fine-tuning SAM. SAMRS (Wang et al., 2024) dataset developed using SAM and existing remote sensing datasets. The powerful feature extraction capability of large vision models can better assist agricultural disease detection. Nevertheless, there hasn’t been any information on the use of large vision models in plant disease detection, particularly for classification and severity estimate.

In this study, we constructed the model DINOV2-FCS for leaf disease classification and severity prediction of a variety of fruits based on the DINOV2 large vision model backbone network. The contributions of this study are as follows:

1. We constructed the model DINOV2-FCS for leaf disease classification and severity prediction of a variety of fruits based on the DINOV2 large vision model backbone network. This approach has been shown to have good generalization ability.
2. In order to enhance the training of the model, the leaf and lesion regions in the 2010 images were meticulously labeled.
3. An improvement to the MLP decoder has been proposed, namely Explicit Feature Fusion Architecture (EFFA), which fuses explicit feature information and multilevel feature information and improves the segmentation accuracy of the model.
4. We have proposed Alterable Kernel Atrous Spatial Pyramid Pooling (AKASPP), which fuses contextual and detailed edge information from different sensory fields in order to enhance adaptability to varying sizes and shapes of lesion targets and to align with the edge details of leaves and lesions.
5. We have proposed Class-Patch Feature Fusion Module (C-PFFM), which fuses local detailed feature information from patch tokens and global feature information from class token, resulting in improved classification accuracy of the model.

## 2 Materials and methods

### 2.1 Datasets

**Figure 1:**
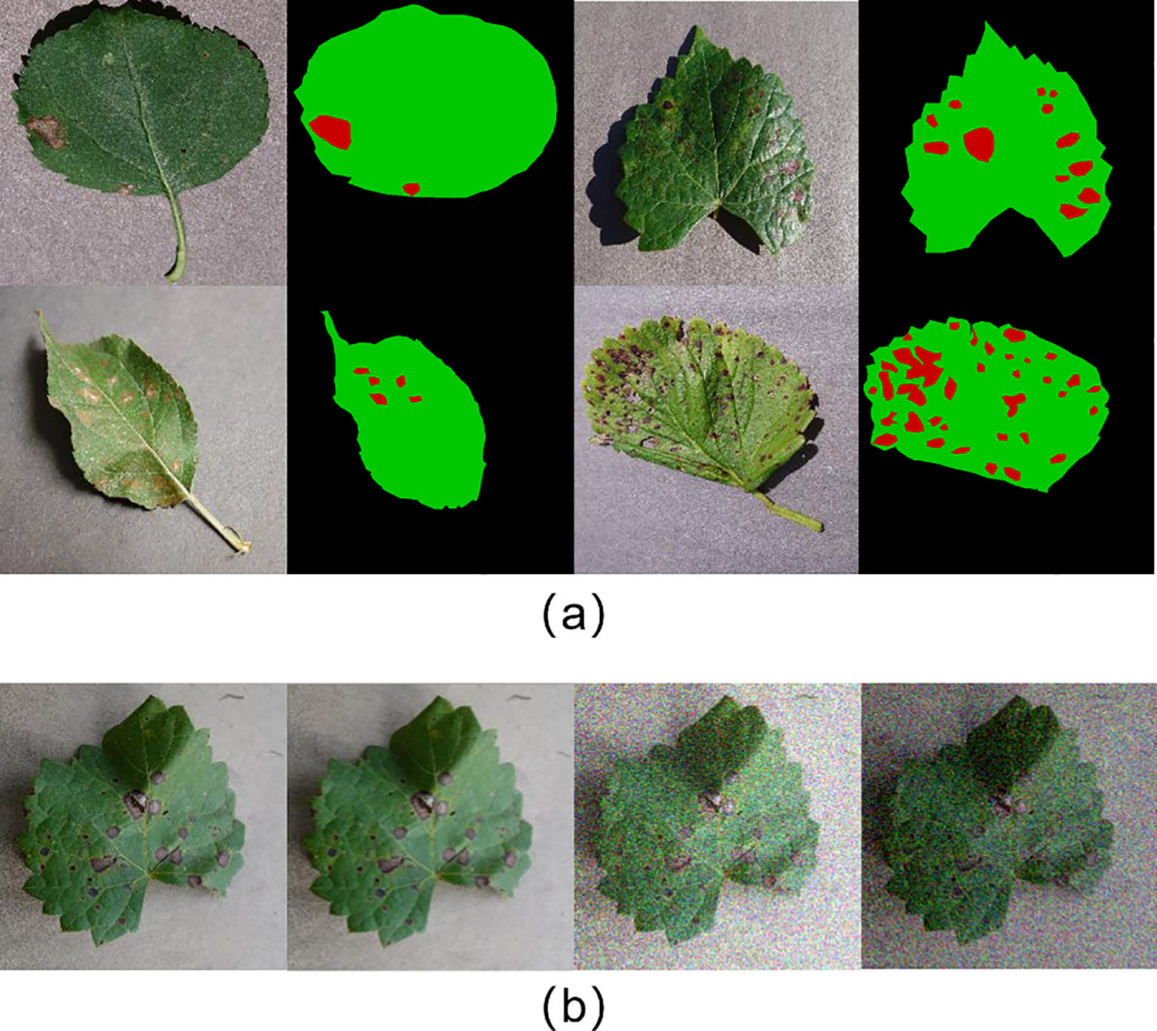
**(A)** Sample dataset annotation; **(B)** Sample data augmentation.

This study collected 2,010 images related to five different fruit foliar diseases: apple black rot, cedar apple rust, grape black measles, grape black rot, and strawberry leaf scorch. These images were obtained from the public PlantVillage dataset (Hughes and Salathé, 2015), which consists of images captured in an indoor laboratory setting and is widely used for crop and plant disease research. We increased the number of images to 8,040 using data augmentation techniques, and all images were accurately labeled. The precise number of images for each disease is presented in **Table 1**. The procedure for processing the dataset was as follows:

1. Uniform image size: The selected images were resized to 512×512 pixels, consistent with the input specifications of the model, by using the resize method of the Image class in the Pillow library (version 10.2.0).
2. Data labeling: The leaf and lesion areas in the images were manually labeled with high accuracy using LabelMe (version 3.16.7). Each image was categorized into three regions: background, leaf, and lesion, represented by black, green, and red, respectively. The labeled images serve as a benchmark for evaluating the accuracy of the segmentation model. **Figure 1A** shows a selection of images from the dataset, alongside their accurately labeled counterparts.
3. Data augmentation: To simulate various lighting conditions and disturbances, data augmentation was applied to the original images by introducing random noise, applying blurring operations, and adjusting brightness. Specifically, NumPy (version 1.24.4) was used to generate Gaussian-distributed noise, which was added to the images. Various blurring algorithms from the OpenCV library (version 4.9.0.80) were applied, and brightness was randomly adjusted using a factor generated by NumPy. This enhanced the diversity of the dataset. **Figure 1B** shows examples of the augmented images.
4. Data splitting: To train the model and evaluate its performance, the dataset was randomly divided into training and test sets with a 7:3 ratio. To ensure reproducibility, the random seed was set to 0.

**Table 1:** Statistics on the number of datasets.

	Apple black rot	Cedar apple rust	Grape black measles	Grape black rot	Strawberry leaf scorch
Original	441	417	419	404	329
Enhanced	1323	1251	1257	1212	987
Total	1764	1668	1676	1616	1316

In practice, calculating the percentage of leaf diseased area is a crucial step in assessing the severity of the disease. Nevertheless, there is as yet no uniform grading scale for the severity of disease. Guided by the experience of experts as well as references to the literature (Wang et al., 2021), this study graded the severity of leaf disease to facilitate a better assessment of model performance. illustrates the grading strategies employed to assess the severity of leaf disease. **Table 2** illustrates the grading strategies employed to determine leaf disease severity.

**Table 2:** Grading strategies for the severity of leaf disease.

Disease grade	Proportion of disease spots in leaves P
Level 0	0
Level 1	0<P ≤ 10%
Level 2	10%<P ≤ 20%
Level 3	20%<P ≤ 40%
Level 4	40%<P ≤ 60%
Level 5	60%<P ≤ 100%

### 2.2 Model structure

In this study, a model, DINOV2-FCS, is constructed based on the DINOV2 large vision model for the purpose of classifying and segmenting diseased leaves of fruits. The DINOv2 model generates generalized visual features through pre-training on a large amount of well-curated data, which are effective across different image distributions and tasks without the need for fine-tuning. The DINOv2-FCS model uses the DINOv2-B (distilled) as the backbone. The DINOv2-B model adopts the ViT-B/14 architecture and consists of 12 consecutive Transformer Blocks. In this study, the classification and segmentation modules are designed separately to accomplish fruit leaf disease classification and severity prediction, respectively, using the features obtained from the backbone.

**Figure 2:**
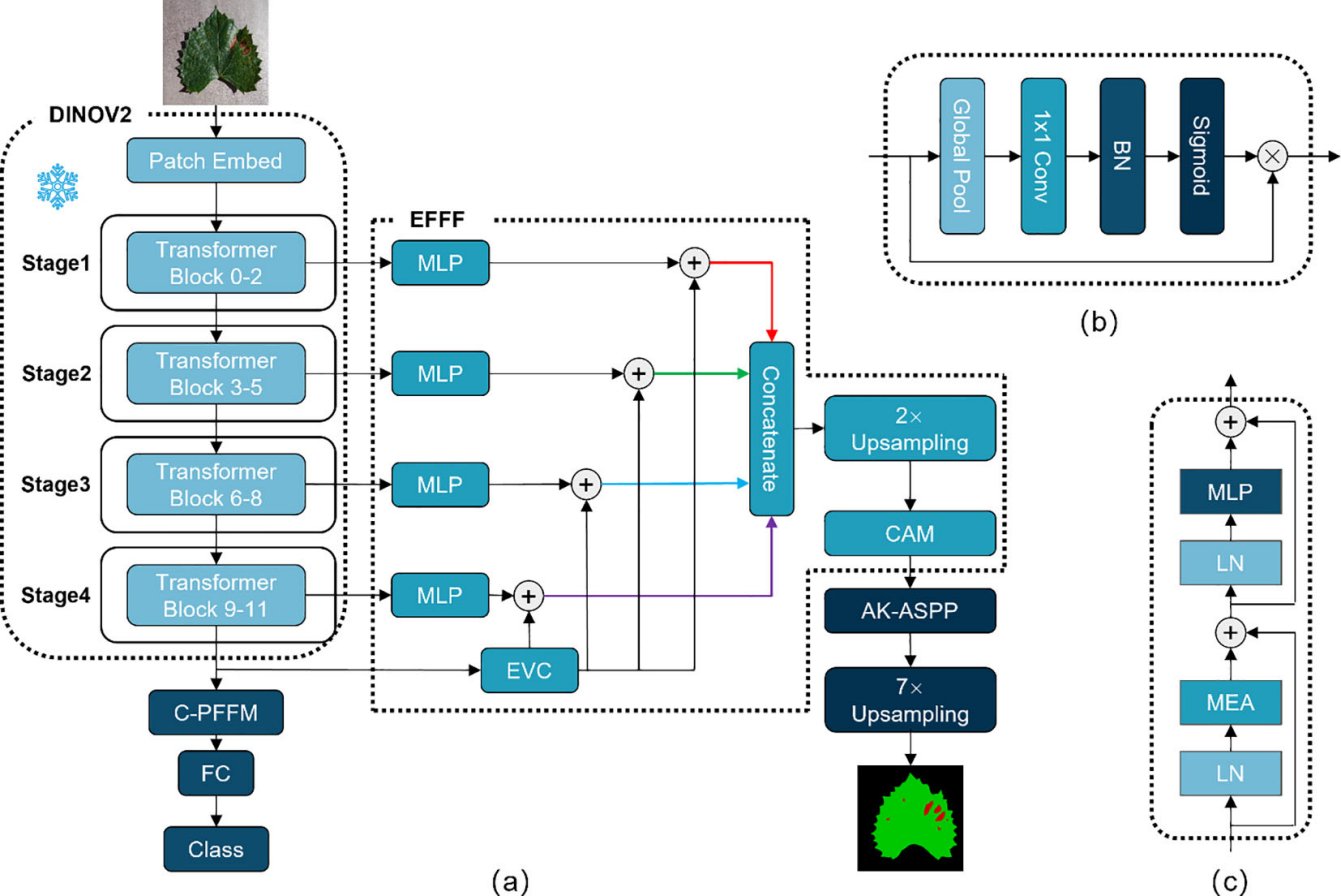
**(A)** Represents the overall structure of DINOV2-FCS; **(B)** represents the structure of CAM; **(C)** represents the structure of Transformer Block.

In the classification module, this study proposes Class-Patch Feature Fusion Module (C-PFFM) as a method of fusing patch tokens and class token for effective feature fusion. C-PFFM is demonstrated to more effectively utilise the features generated by the backbone for disease classification of fruit leaves, and to enhance the model’s classification accuracy. In the segmentation module, the following methods are proposed: Explicit Feature Fusion Architecture (EFFA) and Alterable Kernel Atrous Spatial Pyramid Pooling (AKASPP). EFFA fuses explicit feature information and multilevel feature information. AKASPP fuses contextual information and detailed edge information from different sensory fields. These modules greatly enhance the segmentation performance. The overall model structure is shown in **Figure 2**.

### 2.3 Class-patch feature fusion module

In VIT (Dosovitskiy et al., 2020), the classifier typically inputs the class token to a fully connected layer, after which the classification result is obtained. The advantage of this approach is that the classifier is constructed in a straightforward manner, the number of parameters is minimal. However, utilising the class token as the sole input to the classifier will result in the omission of a substantial quantity of local, detailed feature information. To address this issue, Class-Patch Feature Fusion Module (C-PFFM) is proposed in this study. C-PFFM effectively fuses the local detail feature information of patch tokens and the global feature information of class token, thereby enhancing the model’s classification accuracy. The operation procedure of C-PFFM is illustrated in Equation 1.


(1) $$\{\begin{array}{c} \text{H}=\left(1-\text{α}\right)\cdot \text{avgpool}{\text{X}}_{\text{p}}+\text{α}\cdot {\text{X}}_{\text{c}}\\ \text{α}=\text{CBS}\left(\left(\text{avgpool}{\text{X}}_{\text{p}}+{\text{X}}_{\text{c}}\right)\right) \end{array}$$

**Figure 3:**
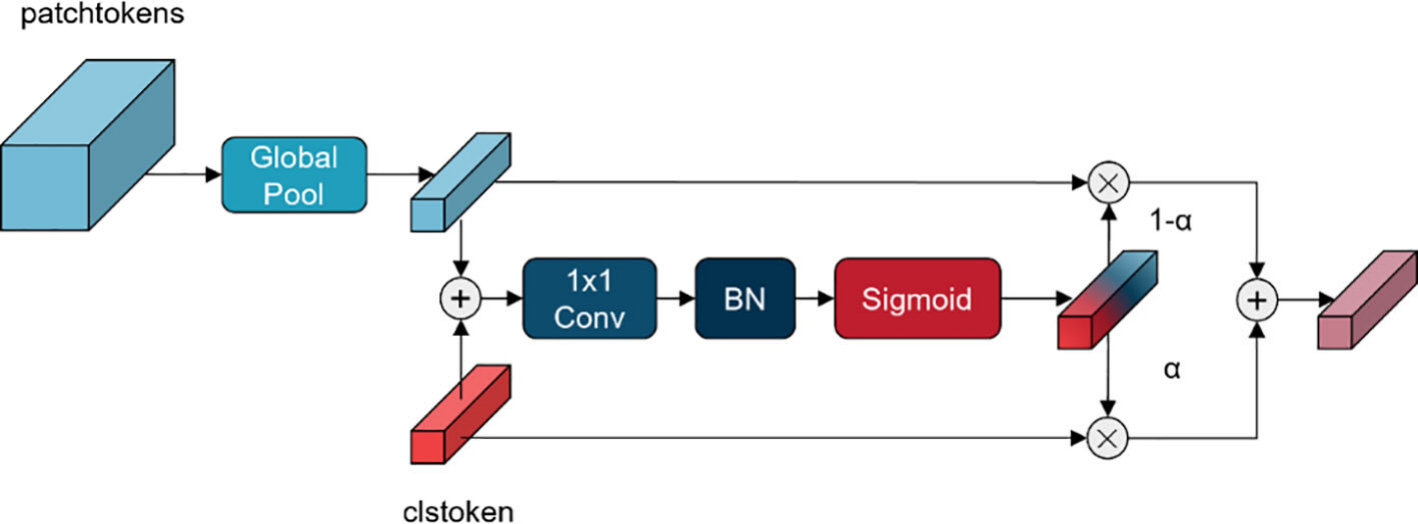
Structure of C-PFFM.


$X_{p}$ denotes patch tokens feature; $X_{c}$ denotes class token feature; avgpool denotes global average pooling operation; *CBS* denotes Convolution + BN + Sigmoid; *X* denotes output feature map;

The final two layers of the backbone feature extraction network, patch tokens feature $X_{p}$ and class token feature $X_{c}$ , are initially identified. Feature *W* is obtained by performing a global average pooling operation on feature $\;X_{p}$ and summing feature $X_{c}$ element by element. The global average pooling operation is illustrated in Equation 2 The feature *W* is then subjected to convolution and BN operations to obtain the channel weights α via the Sigmoid operation. Feature $X_{p}$ is subjected to element-by-element matrix dot-multiplication with the channel weights (1−α) and the feature. The obtained features are subjected to element-by-element summing operation to obtain the patch tokens and class token fusion feature. The structure of C-PFFM is depicted in **Figure 3**.

(2) $${\text{X}}_{\text{avgpool}}=\frac{1}{\text{H}\times \text{W}}\sum_{\text{i}=1}^{\text{H}}\sum_{\text{j}=1}^{\text{W}}{\text{X}}_{(\text{i},\text{j})}$$

*X* denotes the feature map; *H* denotes the height of the feature map; *W* denotes the width of the feature map; $X_{avgpool}$ denotes the feature after global average pooling.

Class token contains long-range global feature information and is often used as input features for classifiers. However, the rich local detailed feature information contained in patch tokens should not be ignored. In particular, in the task of classifying fruit leaf diseases, there is a great similarity between leaf spots of different diseases. If the detailed features are ignored and only the global features are focused on, it will lead to poor classification accuracy of the model. Local information typically encompasses fine structural and local features within an image, whereas global information encompasses the overall context and background knowledge. The effective fusion of the two enables the model to learn a complete and representative feature, thereby enhancing its ability to comprehend the input data and its classification performance.

### 2.4 Explicit feature fusion architecture

SegFormer (Xie et al., 2021) is a straightforward and effective semantic segmentation framework for Transformer. This approach avoids complex decoder design and fuses information from different layers. For semantic segmentation tasks, these feature information are multi-layered global feature information and lack explicit feature information, which makes it difficult to segment some tiny targets. CFPNet (Quan et al., 2023) proposes an Explicit Visual Center (EVC) that focuses on aggregating local corner-region features of an image to enhance the feature representation.In this study, Explicit Feature Fusion Architecture (EFFA) is proposed. The output features from each of the four stages of the DINOV2 backbone are input into the MLP layer to obtain global feature information at multiple levels. Subsequently, the features from the last layer of the DINOV2 backbone are inputted into the EVC to obtain explicit feature information. The explicit feature information is integrated into the global feature information of each layer through a summing operation with the global feature information of multiple layers. Finally, the multilevel feature information is spliced according to the channels and fused by a channel attention. The specific structure of EFFA is illustrated in **Figure 2**.

The image of leaf disease exhibits a multitude of spots of varying sizes. When the model performs segmentation, it is not uncommon that disease spots are incompletely segmented or subtle spots are directly ignored. EVC provides a powerful feature enhancement mechanism for the model. This mechanism enables semantic segmentation models to recognize and localize objects in an image with greater accuracy, particularly in the context of images comprising multiple segmented objects, such as those depicting leaf diseases. The EFFA proposed in this study fuses explicit feature information into global feature information at each level, subsequently fusing multilevel feature information. Multi-level fusion can exploit the complementarity between the underlying and higher-level features to enhance the feature representation. The lowest-level features typically comprise local details and texture information about the image, whereas the highest-level features encompass more abstract semantic information. These multilevel features integrate explicit feature information from EVC.

### 2.5 Alterable kernel atrous spatial pyramid pooling

In fruit leaf images, there are numerous spots with intricate shapes and varying sizes that can significantly impact the segmentation performance of the model. A Pyramid Pooling Module (PPM), comprising a set of pooling blocks with distinct scales, has been proposed in PSPNet (Zhao et al., 2017) based on the concept of pyramid pooling. The PPM provides a comprehensive global representation encompassing the interrelationships between diverse scales and subregions, thereby minimizing the loss of contextual information. DeepLabv2 (Chen et al., 2017a) proposed Atrous Spatial Pyramid Pooling (ASPP) to fuse multi-scale information. In light of this, DeepLabv3 (Chen et al., 2017b) and DeepLabv3+ (Chen et al., 2018) have enhanced the ASPP module, achieving notable outcomes. These modules employ diverse scales of receptive fields for fusion, addressing the issue of varying target sizes in images. However, in the context of fruit leaf disease images, the spot targets are also characterised by intricate shapes and indistinct edges. In this study, a novel approach, AKASPP, is proposed for the fusion of contextual and detailed edge information from different receptive fields. This approach is based on inflated convolution and AKConv (Zhang et al., 2023).

Expansion convolution offers the potential to provide a larger sensory field than conventional convolution. Conventional convolution permits the construction of a receptive field of size K×K when the convolution kernel size is *K*. In contrast, inflated convolution provides a receptive field as illustrated in Equation 3 Alterable Kernel Convolution (AKConv) is a new type of convolutional operation that allows convolution kernels to have an arbitrary number of parameters and an arbitrary sampling shape. In contrast to traditional convolution operations, which are typically constrained to fixed-size windows and fixed sample shapes, AKConv defines the initial position of an arbitrarily sized convolution kernel through a novel coordinate generation algorithm and introduces offsets to accommodate alterations in the target shape. In semantic segmentation tasks, AKConv can facilitate more precise local feature extraction and enhanced edge detail fitting, thereby enhancing the accuracy and detail of segmentation.


(3) $$\text{RF}={\left(\left(\text{r}-1\right)\left(\text{K}-1\right)+\text{K}\right)}^{2}$$

**Figure 4:**
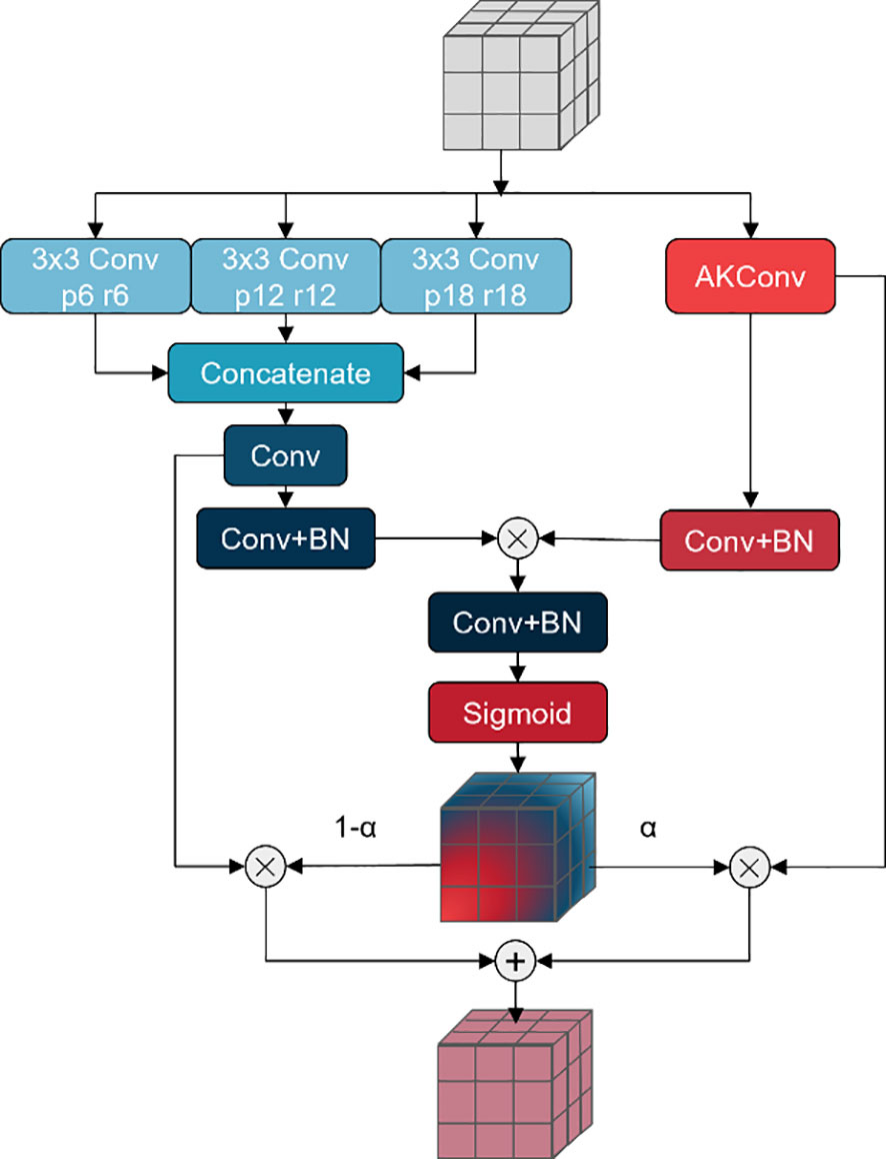
Structure of ASPP.

*RF* denotes the receptive field of the convolution kernel;*r* denotes the expansion rate of the expansion convolution; *K* denotes the convolution kernel size;

In this study, AKASPP is proposed for fruit leaf disease images with complex spot shapes, blurred edges, and different sizes. **Figure 4** illustrates the specific structure of AKASPP. AKASPP is capable of fusing contextual and detailed edge information from different receptive fields. In order to capture features under different receptive fields, expansion convolution with different expansion coefficients is employed. This enables the model to capture a sufficiently wide range of contextual information at different scales, thereby improving the recognition of targets of varying sizes. AKConv permits the convolutional kernel to have an arbitrary sampling shape, which differs from the traditional fixed square sampling shape. This flexibility allows the convolutional kernel to adapt more effectively to the varying shapes of spot targets, and to be sufficiently flexible to capture image features and fit the edge details of leaves and spots, thus improving performance. AKASPP effectively fuses this feature information to better segment different sizes and shapes of spot targets, and to better handle the edge portions of leaves and spots.

### 2.6 Loss functions

**Figure 5:**
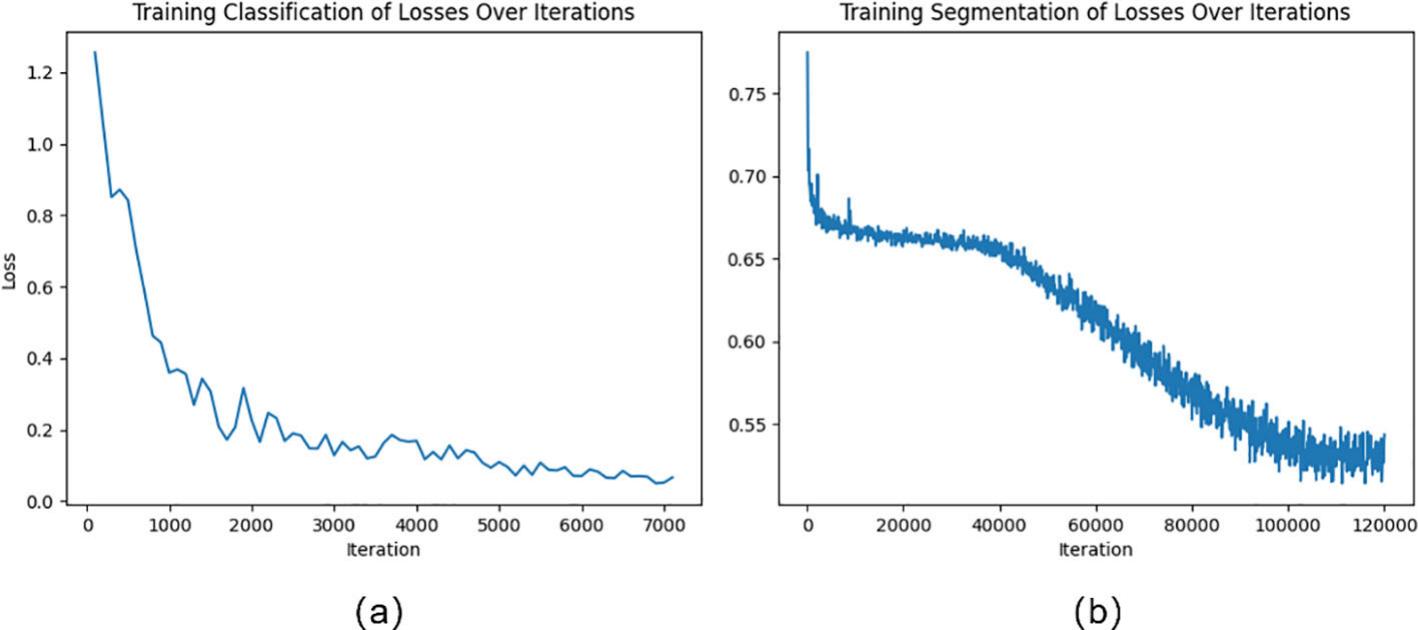
**(A)** Training classification of losses over iterations; **(B)** Training segmentation of losses over iterations.


The cross-entropy loss function is used in this work as the loss function when the classification module is being trained. The cross-entropy loss function is shown in Equation 4. **Figure 5A** illustrates the variation of loss during the training of the classification model. The loss curve gradually becomes smooth after 5000 iterations.

(4) $$\text{L}=\;-\;\frac{1}{\text{N}}\sum_{\text{n}=0}^{\text{N}-1}\text{ylog}\left(\text{p}\right)$$

*L* denotes the indicated cross-entropy loss; *y* denotes the true label of the pixel; *p* denotes the prediction result of the pixel; *N* denotes the number of difficult samples.

Unbalanced categories or a lack of challenging examples are common issues in semantic segmentation tasks, which can impair model performance. In the fruit leaf disease scene segmentation job, for instance, the disease spot category might only cover a minority of the space, but the leaf category might represent the majority. Insufficient performance in predicting other categories may result from the model’s training primarily focusing on the leaf category. Online Hard Sample Mining (OHEM) can assist the model in focusing on difficult and rare samples, thereby improving overall performance (Shrivastava et al., 2016). In this study, the cross-entropy loss function of the semantic segmentation module includes OHEM. The loss function in this study is shown in Equations 5–7. **Figure 5A** illustrates the variation of loss during the training of the segmentation model. The loss curve gradually becomes smooth after 100000 iterations.


(5) $${\text{l}}_{\text{CE}}=\;-\text{ylog}\left(\text{p}\right)$$

(6) $${\text{l}}_{\text{Hard}}=\;{\text{l}}_{\text{CE}},\;{\text{l}}_{\text{CE}}>0.7$$

(7) $${\text{L}}_{\text{ohemCE}}=\;\frac{1}{\text{M}}\sum_{\text{m}=0}^{\text{M}-1}l_{\text{Hard}}$$

$l_{CE}$ denotes cross-entropy loss; *y* denotes the true label of the pixel; *p* denotes the prediction result of the pixel; $l_{Hard}$ denotes the loss of difficult samples; $L_{ohemCE}$ denotes the loss function in the OHEM combined with the cross-entropy loss function; *M* denotes the number of difficult samples.

## 3 Experimental results

### 3.1 Disease classification results

**Figure 6:**
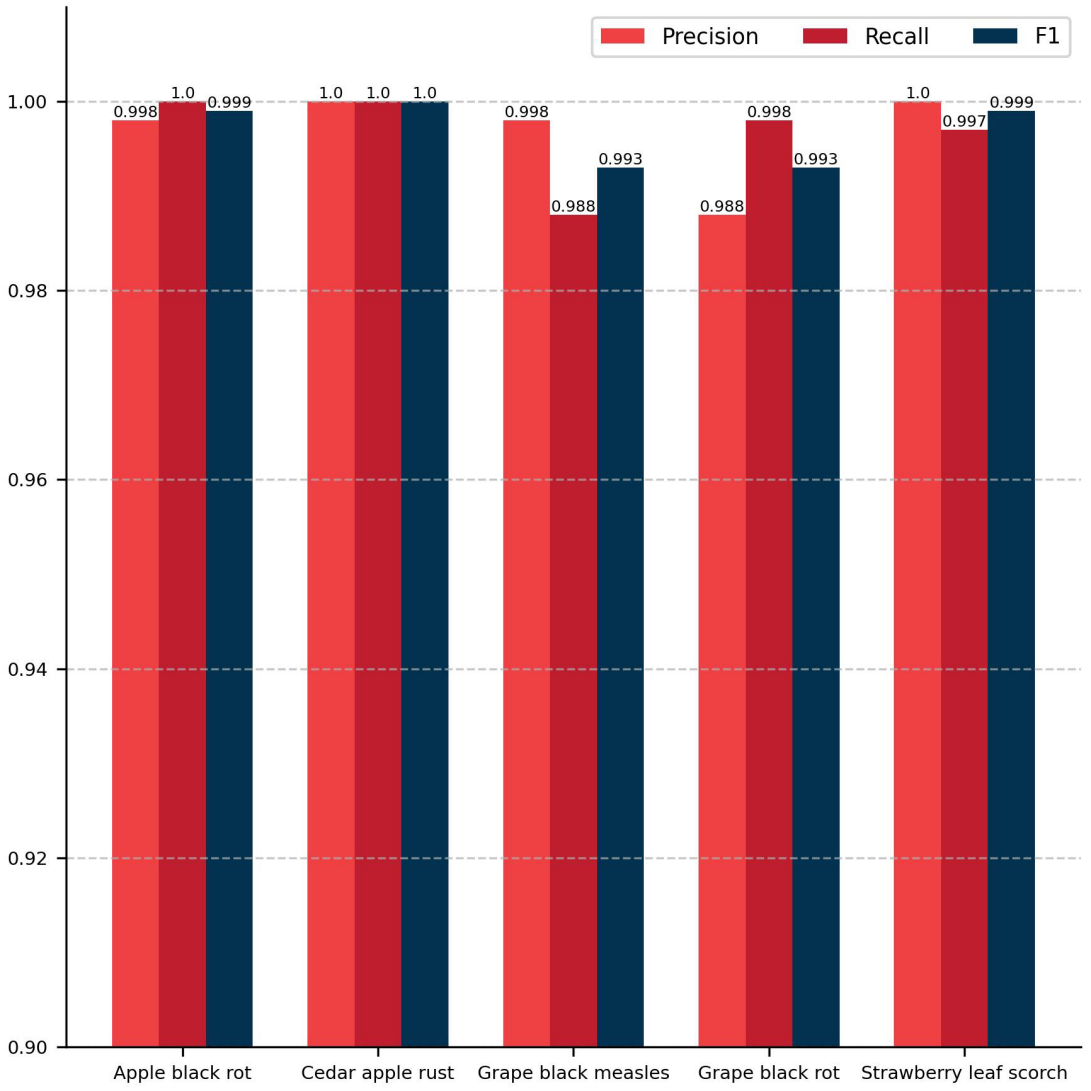
Histogram of classification results.

**Figure 7:**
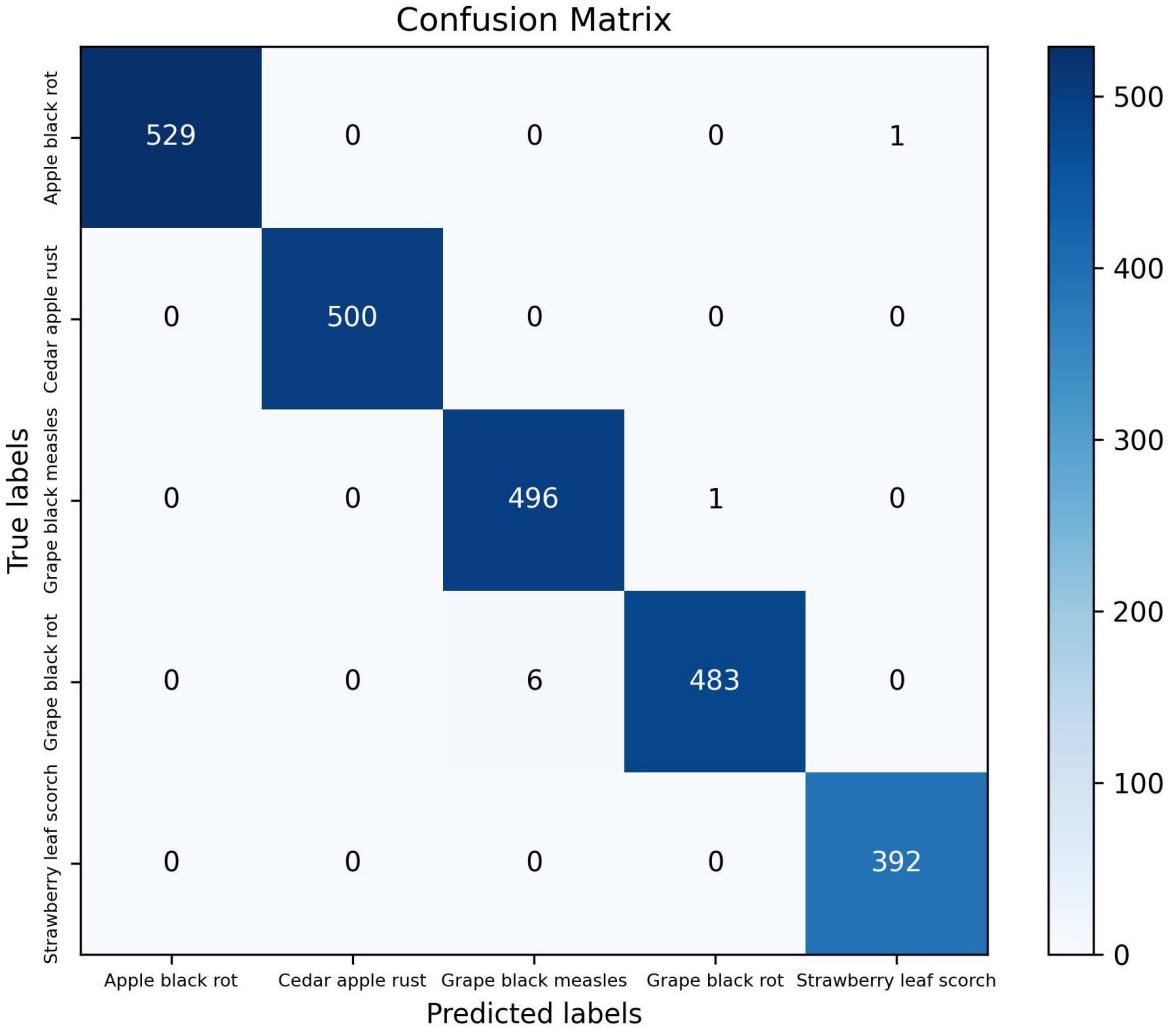
Confusion matrix for classification results.

The classification module of the model proposed in this study achieved a ACC of 99.67% and a Macro F1 of 99.67% on the test set. **Figure 6** presents the evaluation results of five distinct plant disease classification algorithms, including precision, recall, and F1 score. The diseases are presented from left to right in the following order: apple black rot, cedar apple rust, grape black measles, grape black rot, and strawberry leaf scorch. For each disease, the values of the three evaluation metrics are nearly identical, indicating that the model proposed in this study has high accuracy in recognizing these specific plant diseases. **Figure 7** depicts a confusion matrix plot for the purpose of evaluating the performance of a classification model. The x-axis represents the predicted labels, the y-axis represents the true labels, the diagonal of the matrix represents the number of correct disease predictions, and the rest of the matrix represents misclassifications. As illustrated in the figure, the model exhibited a high degree of accuracy in classifying diseased leaves in the test set, correctly identifying the vast majority of samples. Only a small number of samples were misclassified. For instance, in the sample pertaining to apple black rot, there were 529 correctly classified samples, with only 1 misclassified as strawberry leaf scorch. Among the samples of grape black rot, 483 were correctly classified, while 6 were misclassified as grape black measles due to the high degree of similarity between the two grape diseases. Nevertheless, the model achieved satisfactory results. In conclusion, the DINOV2-FCS proposed in this study is an excellent tool for the classification of fruit leaf diseases.

### 3.2 Semantic segmentation results

**Figure 8:**
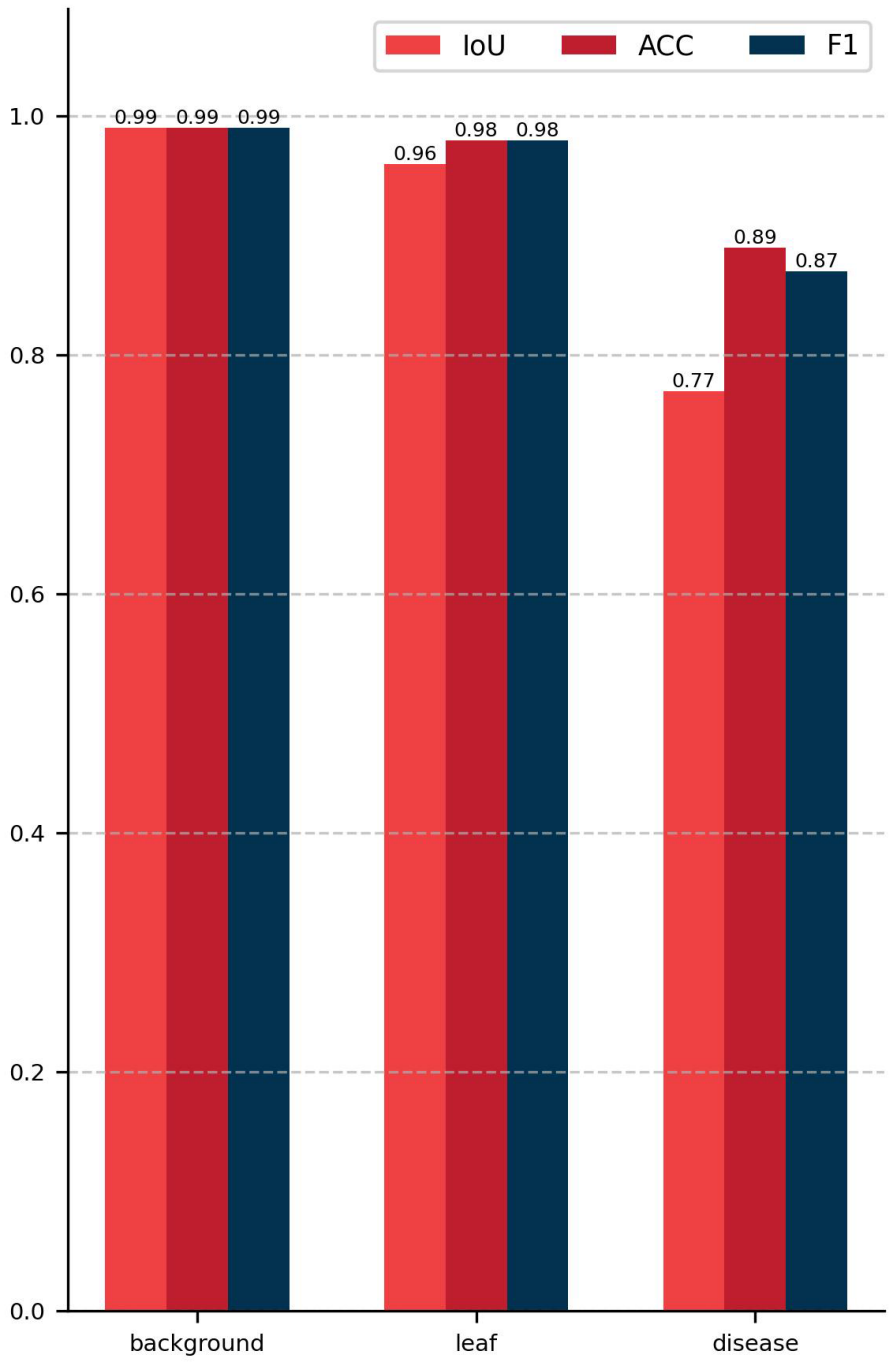
Histogram of semantic segmentation results.

**Figure 9:**
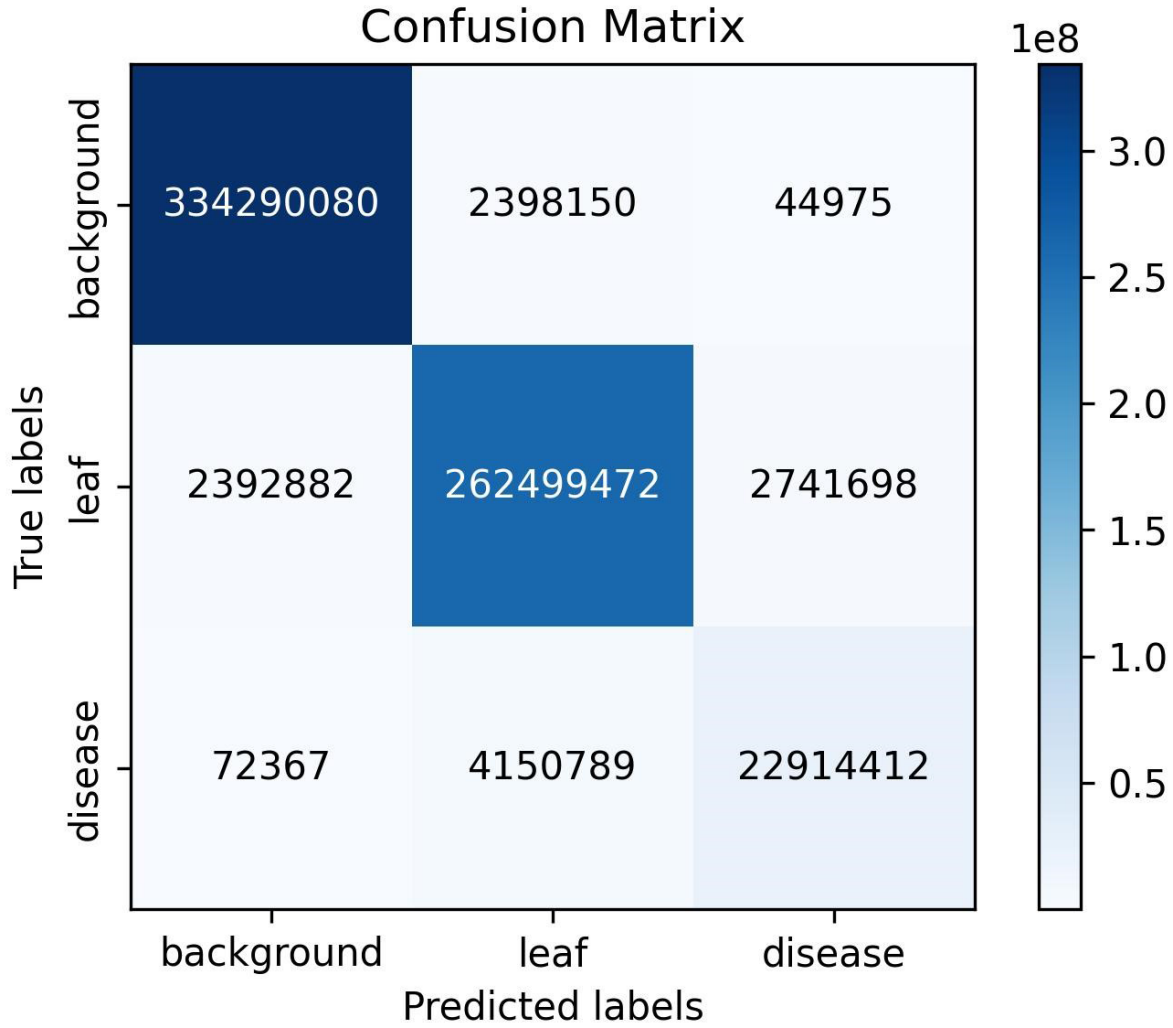
Confusion matrix of semantic segmentation results.

The semantic segmentation module of the model proposed in this study achieved a mIoU of 90.29, a PA of 98.13%, and a Macro F1 of 94.61% on the test set. **Figure 8** presents the outcomes of the evaluation of the semantic segmentation algorithm for three categories, including three evaluation metrics: IoU, PA, and F1. The IoU, PA, and F1 for the background category are 0.99, the leaf category is 0.96, 0.98, and 0.98, respectively, and the disease category is 0.77, 0.89, and 0.87, respectively. The data in **Figure 8** indicates that the background category achieved the best evaluation results, the leaf category was the next best, and the disease category had the worst evaluation results. This phenomenon can be attributed to the fact that in images where the background and leaves tend to occupy the majority of pixels, the disease only occupies a small number of pixels. This results in a significant imbalance in the number of samples, which impedes the network’s ability to learn sufficient information about the pixels in the disease category. As illustrated in **Figure 9**, the vast majority of pixels are correctly categorized, with only a small number of pixels not being correctly classified. The figure also demonstrates that the disease category has a relatively small number of pixels compared to the other categories. In conclusion, the DINOV2-FCS proposed in this study demonstrates satisfactory performance in the segmentation of leaf diseases.

### 3.3 Results of leaf disease severity prediction

**Figure 10:**
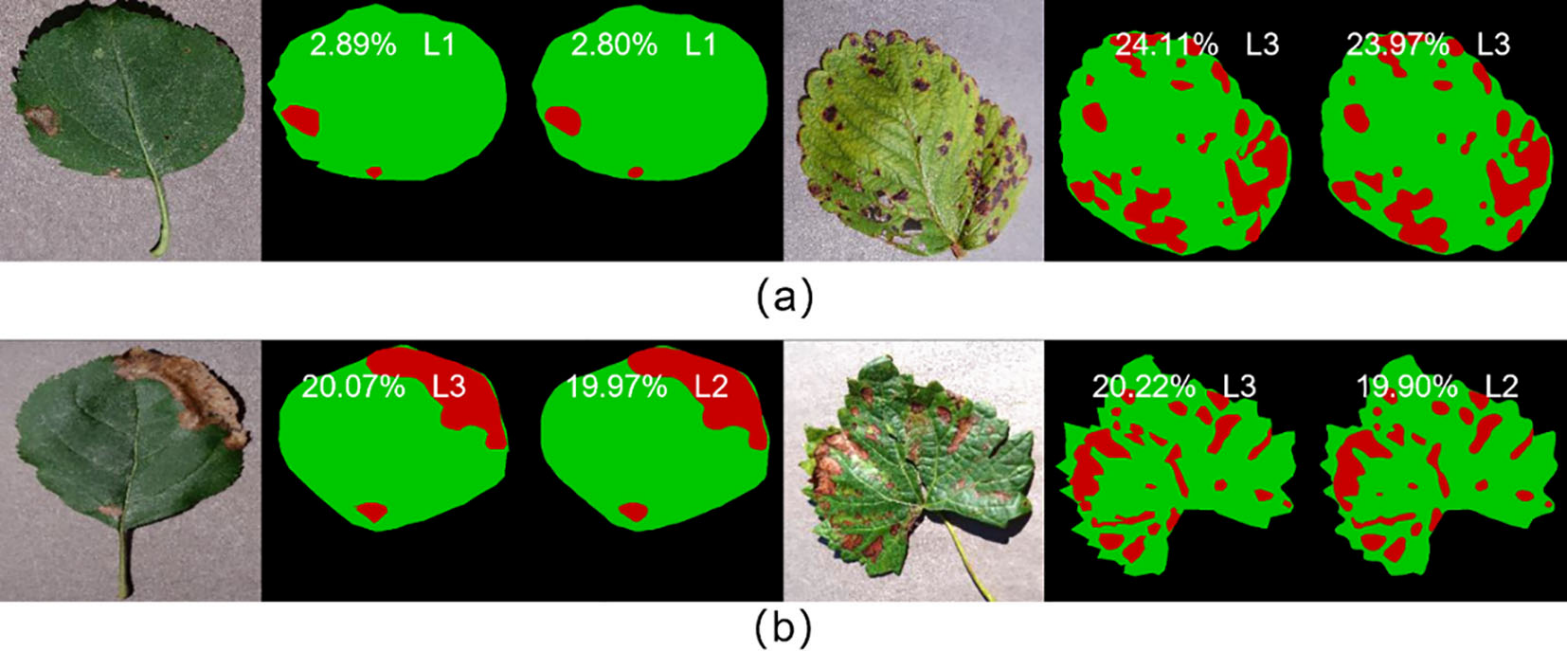
**(A)** Represents the samples with correct prediction of leaf disease severity grading; **(B)** represents the samples with incorrect prediction of leaf disease severity grading.

In this study, the fruit leaf disease severity was categorized into five classes. The model proposed in this work exhibited 95.68% accuracy in grading prediction on the test set. As illustrated in **Figure 10**, the model employed in this study demonstrated satisfactory performance in predicting the severity of fruit leaf disease. The proximity between the ratio of diseased spot area to total leaf area predicted by the model and the true label was high, with a difference of less than 0.40% observed even in individual samples where the prediction grading was erroneous. Consequently, the model in this study exhibited satisfactory capacity for the measurement of fruit leaf disease severity.

### 3.4 Comparison of other models

In order to evaluate the performance of the classification module of DINOV2-FCS proposed in this study, four state-of-the-art mainstream classification models, namely ResNet (He et al., 2016), VIT, ConvNext (Liu et al., 2022), and Swin (Liu et al., 2021), have been selected for comparison. The evaluation metrics chosen are ACC, Macro F1, and Params. It should be noted that these models freeze the backbone network during training as DINOV2-FCS.

**Figure 11:**
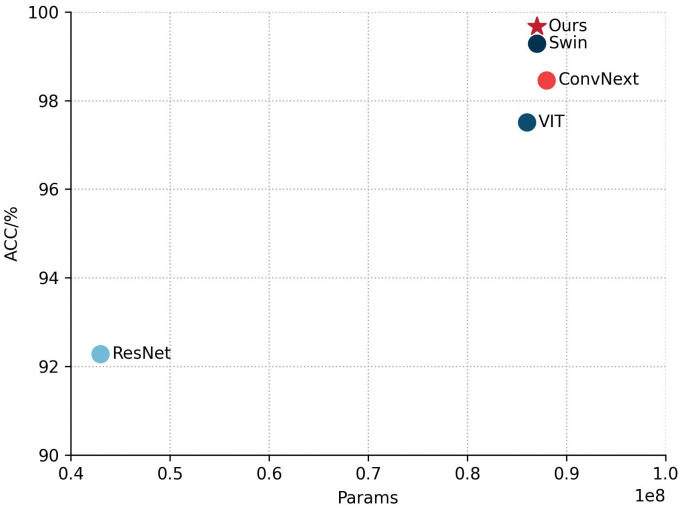
Scatterplot of ACC and Params for different models.

**Table 3** shows a comparison of the performance of different models on the fruit leaf disease classification task, where our model performs best with 99.67% ACC and Macro F1, and the same number of covariates is about 0.87 × 10^8^. This indicates that the model proposed in this study achieves top level accuracy and F1 score while maintaining relatively compact parameter scales, outperforming all the benchmark models compared. **Figure 11** shows scatter plots of the ACC and Params counts of the different models, with five points representing five different models. By observing the position of the points in the plot, we can see that our model performs very well in terms of Params and ACC, outperforming the other four models. In summary, the classification module of DINOV2-FCS proposed in this study is the most outstanding in terms of performance, not only achieving the highest accuracy and F1 score, but also comparable to the Swin base version in terms of model complexity, showing a very high level of efficiency and optimization.

**Table 3:** Classification performance of different models.

Model	ACC/%	Macro F1/%	Params
ResNet101	92.28	92.42	0.43×10^8^
VIT(Base)	97.51	95.57	0.86×10^8^
ConvNext(Base)	98.46	98.50	0.88×10^8^
Swin(Base)	99.29	99.31	0.87×10^8^
Ours	99.67	99.67	0.87×10^8^

In order to evaluate the performance of the semantic segmentation module for DINOV2-FCS proposed in this study, we selected seven advanced mainstream semantic segmentation models, namely FCN (Long et al., 2015), Deeplabv3+, SETR (Zheng et al., 2021), SegMenter (Strudel et al., 2021), SegFormer, MaskFormer (Cheng et al., 2021) and Mask2Former (Cheng et al., 2022). The comparison is performed. The evaluation metrics chosen are mIoU, PA, Macro F1 and Params. It should be noted that these models are trained with and without backbone network freezing, respectively, and DINOV2-FCS proposed in this study freezes the backbone network during training.

**Figure 12:**
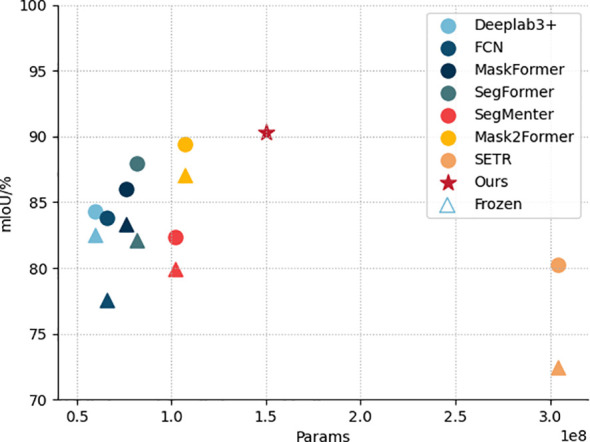
Scatterplot of mIoU and Params for different models.

**Table 4** shows the performance comparison of several semantic segmentation models on different evaluation metrics, where asterisks denote the freezing of the backbone network, and the model DINOV2-FCS proposed in this study, which leads in all metrics, with 90.29% of mIoU, 94.61% of Macro F1, 98.13% of PA, and 1.50 × 10^8^ of Params, reflecting the effectiveness and progress of the model design. **Figure 12** shows the scatter plots of mIoU and Params for different models, where each color represents one model. In the models, circles represent training without freezing the backbone network, triangles represent training with freezing the backbone network, and pentagram represents the model proposed in this study. By observing the position of the pentagram in the figure, we can see that our model outperforms the other models in terms of Params and mIoU. In the case of freezing the backbone network, all the other models show performance degradation, but the model proposed in this study still outperforms all the models in terms of performance in the case of freezing the backbone network. In summary, this study proposes that the semantic segmentation module of DINOV2-FCS has the best performance, not only achieving the highest mIoU, Macro F1 and PA. Meanwhile, the Params is smaller than that of SETR, which demonstrates its superiority in semantic segmentation tasks.

**Table 4:** Segmentation performance of different models.

Model	mIoU/%	Macro F1/%	PA/%	Params
FCN(R101)	83.83	90.30	96.79	0.66×10^8^
FCN(R101)*	77.53	85.34	95.46	0.66×10^8^
Deeplabv3+(R101)	84.32	90.66	96.86	0.60×10^8^
Deeplabv3+(R101)*	82.48	89.31	96.49	0.60×10^8^
SETR(VIT-L)	80.28	87.60	96.06	3.04×10^8^
SETR(VIT-L)*	72.42	80.47	94.55	3.04×10^8^
SegMenter(VIT-B)	82.38	89.23	96.47	1.02×10^8^
SegMenter(VIT-B)*	79.92	87.37	95.84	1.02×10^8^
SegFormer(MIT-B5)	87.96	93.15	97.59	0.82×10^8^
SegFormer(MIT-B5)*	82.11	89.01	96.46	0.82×10^8^
MaskFormer(R152)	86.03	91.88	97.12	0.76×10^8^
MaskFormer(R152)*	83.34	89.96	96.60	0.76×10^8^
Mask2Former(SwinB)	89.39	94.07	97.81	1.07×10^8^
Mask2Former(SwinB)*	87.10	92.60	97.34	1.07×10^8^
Ours*	90.29	94.61	98.13	1.50×10^8^

(“*” indicates that the backbone network was frozen during model training.)

**Figure 13:**
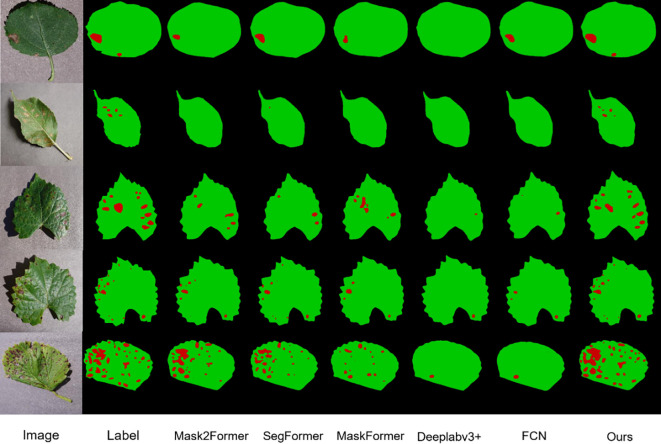
Segmentation effect of different models.

In **Figure 13**, the models Mask2Former, SegFormer, Maskforme, Deeplabv3+, and FCN, which exhibited superior performance on the dataset, are presented for comparison with the models in this study. It can be observed that although they also achieved satisfactory results, instances were identified where a considerable number of lesions were not entirely segmented, and even numerous fine lesions were not detected. In contrast, the model proposed in this study is not subject to the same limitations when segmenting fruit leaf disease images, and the overall segmentation effect is superior. This is due to the powerful feature extraction capability of DINOV2 and the improvement of the model by the characteristics of the disease spots in this study.

## 4 Discussions

### 4.1 Effectiveness of DINOV2 backbone network

**Figure 14:**
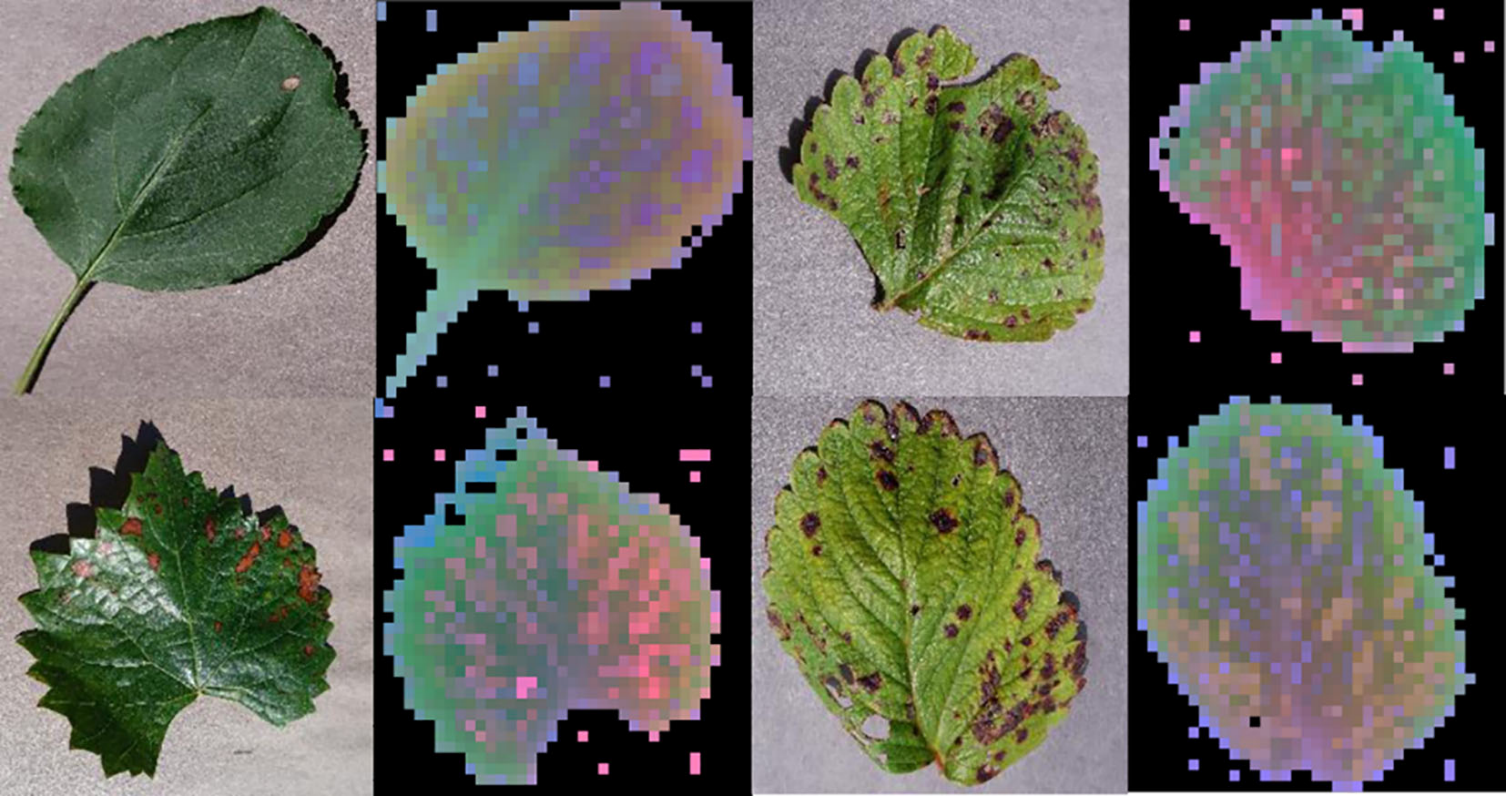
Visualization of principal component analysis of DINOV2 generated features.

In order to verify the feature extraction capability of the DINOV2 trunk feature extraction network, we performed principal component analysis (PCA) on the patch features extracted by the DINOV2 model. The features of the input image extracted by this model were subjected to PCA dimensionality reduction in order to map the high-dimensional features to the three-dimensional space. The background and foreground portions of the image were then judged based on the results of PCA, with the principal components of the foreground portion being renormalized in order to highlight them. The visualization facilitates comprehension of the feature extraction effect of the DINOv2 model on the image, as well as the structure and distribution in the feature space after dimensionality reduction by PCA. As illustrated in **Figure 14**, the DINOV2 model exhibits high performance in distinguishing between foreground and background regions in the image, and in delineating the boundaries of the main objects in the picture. Moreover, the DINOV2 backbone feature extraction network has not encountered these images prior to extraction, and the backbone feature extraction network remains fixed throughout the training process of this working model. This indicates that the DINOV2 backbone feature extraction network is well-suited for the extraction of features in images of fruit leaves affected by disease.

### 4.2 Effectiveness of C-PFFM

In order to verify the effectiveness of the C-PFFM proposed in this study, ablation experiments are designed to test the effectiveness of the C-PFFM. In the classification module, DINOV2 is used as the backbone feature extraction network in the first group, and one fully connected layer is used as the classifier. The second experimental group, which combined C-PFFM, was constituted on the basis of the first group. The evaluation metrics used are ACC, Macro F1, and Params. The results of the ablation experiments are presented in **Table 5**. We performed multiple replicated experiments on the proposed models. For the classification model, we selected one of the most important metrics, ACC, to conduct an ANOVA, and the results show that the p-value is 3.8×10^-4^, and the difference is statistically significant.

**Table 5:** Classification module ablation experiment.

	C-PFFM	ACC/%	Macro F1/%	Params
First group	×	97.80	97.86	0.86×10^8^
Second group	√	99.67	99.67	0.87×10^8^

As illustrated in the accompanying table, the C-PFFM proposed in this study has demonstrably enhanced the model’s predictive capabilities. The benchmark model in the first group achieved an ACC of 97.80%, a Macro F1 of 97.86%, and a Params value of 0.86 × 10^8^. In the second group, the C-PFFM was introduced, which represents an effective fusion of local detail feature information from the patch tokens and global feature information from the class token. This resulted in an enhancement of the classification accuracy of the model. The model achieved an ACC of 99.67%, a Macro F1 of 99.67% and 0.87×10^8^ for the Params. The model’s accuracy was significantly enhanced with the same number of parameters. This is due to the fact that in the initial set of experiments, only the class token was utilized as input to the fully connected layer, and the class token contains global feature information over long distances. In the context of classifying fruit leaf diseases, there is a notable similarity between the leaf spots of different diseases. This can result in suboptimal model classification accuracy if detailed features are overlooked and only global features are prioritized. The C-PFFM proposed in this study effectively integrates these features, leading to a notable performance improvement.

### 4.3 Effectiveness of segmentation modules

In order to ascertain the efficacy of the proposed enhancements to the segmentation module in this study, ablation experiments have been designed to assess the impact of these improvements. In the segmentation module, the DINOV2 network is employed as the backbone feature extraction network in the first group, resulting in the generation of a segmented image through up-sampling using the MLP decoder. The second experimental group, which combined EFFA, was constituted on the basis of the first group. The third experimental group, which combined AKASPP, was constituted on the basis of the first group. The fourth experimental group, which combined EFFA and AKASPP, was constituted on the basis of the first group. The evaluation indexes are mIoU, Macro F1, PA, and Params. The results of the ablation experiments are presented in **Table 6**. We performed multiple replicated experiments on the proposed models. For the semantic segmentation model, we selected one of the most important metrics, MIoU, for ANOVA, and the results showed that the p-value was 1.5×10-5, and the difference was statistically significant.

**Table 6:** Segmentation module ablation experiment.

	EFFA	AKASPP	mIoU/%	Macro F1/%	PA/%	Params
First group	×	×	84.56	90.81	96.98	0.90×10^8^
Second group	√	×	88.46	93.45	97.77	1.37×10^8^
Third group	×	√	89.22	93.94	97.93	1.03×10^8^
Fourth group	√	√	90.29	94.61	98.13	1.50×10^8^

As illustrated in the accompanying table, the proposed enhancements to the segmentation module have demonstrably enhanced the model’s performance. The mIoU of the benchmark model in the first group reached 84.56%, the Macro F1 reached 90.81%, the PA reached 96.98%, and the Params was 0.90 × 10^8^. The incorporation of the EFFA into the second group, which fuses explicit feature information with multilevel feature information, resulted in an mIoU of 88.46%, a Macro F1 of 93.45%, and a PA of 97.77%. Additionally, the Params increased to 1.37 × 10^8^. Despite an increase in the number of parameters, there was a notable improvement in accuracy, with an increase of 3.9% in the mIoU. This is attributed to the incorporation of explicit feature information from EVC into multilevel features, which enables the model to simultaneously consider the details and semantic information, thereby enhancing its ability to comprehend the image content. The addition of AKASPP to the third group enables the fusion of contextual and detail edge information from different sensory fields, resulting in an mIoU of 89.22%, a Macro F1 of 93.94%, and a PA of 97.93%, with a Params of 1.37 × 10^8^. With a modest increase in the Params, the mIoU was enhanced by 4.66%, which can be attributed to the fact that the fruit leaf disease image spots exhibit complex shapes, fuzzy edges, and varying sizes. AKASPP effectively fuses contextual and detailed edge information from disparate sensory fields, enabling more precise segmentation of diverse spot targets of varying sizes and shapes, as well as enhanced processing of leaf and spot edge components. The fourth group incorporated both EFFA and AKASPP, based on the findings of the first group. This resulted in an mIoU of 90.29%, a Macro F1 of 94.61%, a PA of 98.13%, and a Params of 1.50×10^8^, which achieved the optimal performance.

### 4.4 Validation of model generalization capabilities

**Figure 15:**
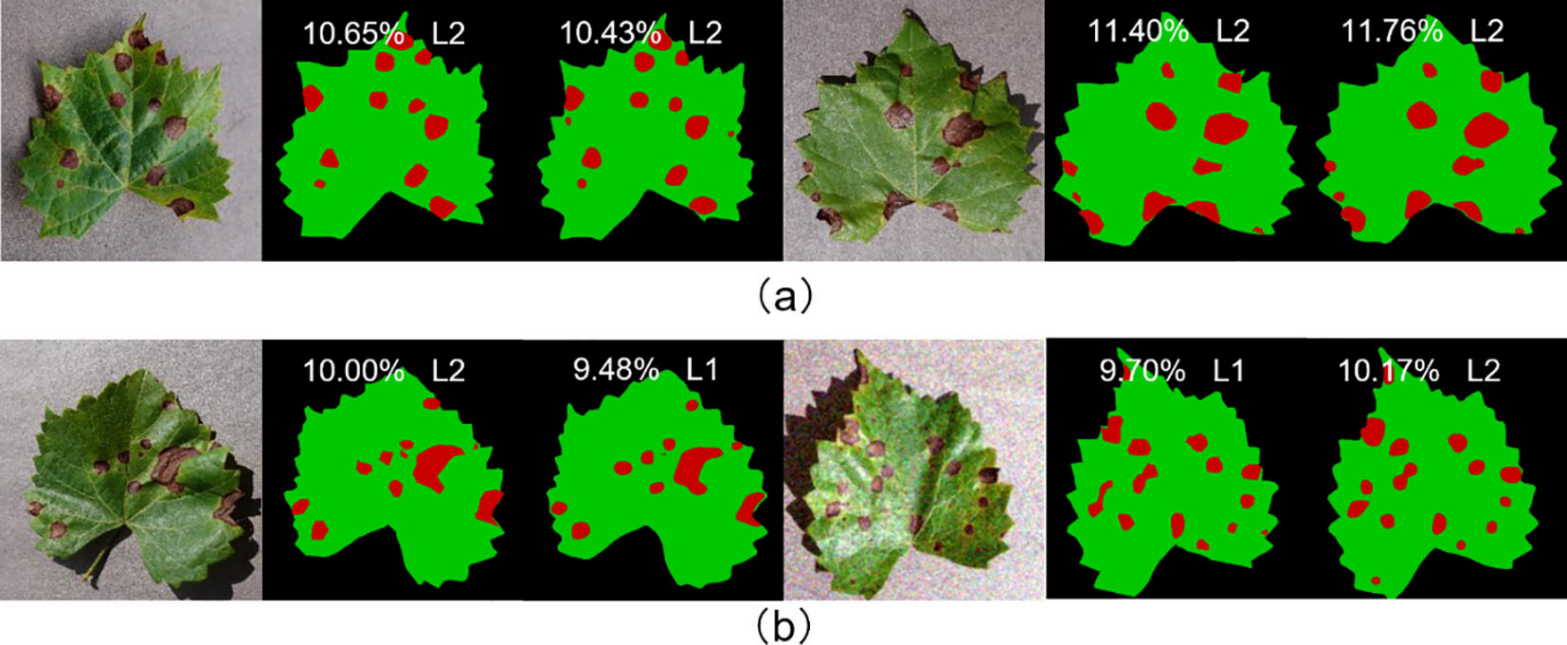
**(A)** Represents the samples with correct prediction of leaf disease severity grading; **(B)** represents the samples with incorrect prediction of leaf disease severity grading.

In order to assess the model’s ability to generalize, four of the five labeled fruit leaf disease datasets were used as the training set, with one dataset reserved for the test set. The training set includes images of four diseases: apple black rot, cedar apple rust, grape black measles, and strawberry leaf scorch. The test set includes images of grape black rot. The semantic segmentation module achieved an mIoU of 83.95% and the fruit leaf disease severity reached the grading accuracy of 95.24%, thereby verifying the strong generalization ability of the model. As illustrated in **Figure 15**, the model exhibited strong generalization ability. The model demonstrated effective performance in segmenting diseases that had never been encountered before. The proximity between the ratio of diseased area to total leaf area predicted by the model and the true label was high, and the difference was minimal even in individual samples where the prediction was incorrectly graded.

## 5 Conclusion

In this study, we constructed the model DINOV2-FCS for leaf disease classification and severity prediction of a variety of fruits based on the DINOV2 large vision model backbone network. The model addresses the shortcomings of current models in disease severity prediction, namely their lack of accuracy and limited generalizability. DINOV2-FCS employs DINOv2-B (distilled) as the backbone feature extraction network to enhance the extraction of features from fruit diseased leaf images. In the context of fruit leaf disease classification, where the leaf spots of different diseases exhibit considerable similarity and the loss of detail information is a significant issue, we propose Class-Patch Feature Fusion Module (C-PFFM), which fuses the local detail feature information of patch tokens and the global feature information of class token. This results in an improvement in the classification accuracy of the model. In light of the fact that the model frequently fails to complete the segmentation of lesions, including those that are subtle, and that lesions are often ignored entirely, we have enhanced the MLP decoder and proposed EFFA, which fuses explicit feature information and multi-level feature information. This has led to an improvement in the segmentation accuracy of the model. Furthermore, we have proposed AKASPP, which fuses contextual information and detailed edge information from different sensory fields, thereby enabling better adaptation to the varying sizes and shapes of lesion targets and the edge details of leaves and lesions. To verify the accuracy and generalizability of the model, two sets of experiments were conducted. First, the labeled leaf disease dataset of five fruits was randomly divided. The trained model exhibited an accuracy of 99.67% in disease classification, an mIoU of 90.29%, and an accuracy of 95.68% in disease severity classification. These results demonstrate superior performance compared to other state-of-the-art models. In the generalizability experiment, four disease data sets were used for training and one for testing. The mIoU of the trained model reached 83.95%, and the accuracy of disease severity grading was 95.24%. The strong generalization ability of the model was verified. The subsequent stage of the process involves the augmentation of the dataset with respect to both species diversity and environmental diversity, thereby aligning it with more realistic scenarios. Furthermore, the model was tested on an NVIDIA GeForce RTX 3090 graphics card, achieving an inference speed of 21.56 frames per second (F/S). The next phase of the project will focus on refining the model to enable its deployment on mobile devices. This will support agricultural workers by assisting with disease identification in the field.

## Data Availability

The raw data supporting the conclusions of this article will be made available by the authors, without undue reservation.
